# Stresses and strains on the human fetal skeleton during development

**DOI:** 10.1098/rsif.2017.0593

**Published:** 2018-01-24

**Authors:** Stefaan W. Verbruggen, Bernhard Kainz, Susan C. Shelmerdine, Joseph V. Hajnal, Mary A. Rutherford, Owen J. Arthurs, Andrew T. M. Phillips, Niamh C. Nowlan

**Affiliations:** 1 Department of Bioengineering, Imperial College London, London, UK; 2 Department of Computing, Imperial College London, London, UK; 3 Department of Civil and Environmental Engineering, Imperial College London, London, UK; 4 Department of Radiology, Great Ormond Street Hospital, London, UK; 5 Department of Biomedical Engineering & Centre for the Developing Brain, School of Biomedical Engineering and Imaging Science, Kings College London, London, UK; 6 Department of Perinatal Imaging and Health & Centre for the Developing Brain, School of Biomedical Engineering and Imaging Science, Kings College London, London, UK; 7 UCL Great Ormond Street Institute of Child Health, London, UK

**Keywords:** musculo-skeletal development, joint biomechanics, cine-MRI, biomechanical stimuli, finite element analysis

## Abstract

Mechanical forces generated by fetal kicks and movements result in stimulation of the fetal skeleton in the form of stress and strain. This stimulation is known to be critical for prenatal musculoskeletal development; indeed, abnormal or absent movements have been implicated in multiple congenital disorders. However, the mechanical stress and strain experienced by the developing human skeleton *in utero* have never before been characterized. Here, we quantify the biomechanics of fetal movements during the second half of gestation by modelling fetal movements captured using novel cine-magnetic resonance imaging technology. By tracking these movements, quantifying fetal kick and muscle forces, and applying them to three-dimensional geometries of the fetal skeleton, we test the hypothesis that stress and strain change over ontogeny. We find that fetal kick force increases significantly from 20 to 30 weeks' gestation, before decreasing towards term. However, stress and strain in the fetal skeleton rises significantly over the latter half of gestation. This increasing trend with gestational age is important because changes in fetal movement patterns in late pregnancy have been linked to poor fetal outcomes and musculoskeletal malformations. This research represents the first quantification of kick force and mechanical stress and strain due to fetal movements in the human skeleton *in utero*, thus advancing our understanding of the biomechanical environment of the uterus. Further, by revealing a potential link between fetal biomechanics and skeletal malformations, our work will stimulate future research in tissue engineering and mechanobiology.

## 1. Introduction

Fetal movements during pregnancy have long been of interest to the scientific and medical communities, as well as to society at large. In humans, the first fetal movement that is observed is a bending of the head and neck at 10 weeks [1], followed by a full range of movements (whole-body movements, limb movements, breathing and stretching) that occur regularly from 15 weeks [2]. Maternal sensation of these movements usually begins between 16 and 18 weeks [2]. While the number of fetal movements is thought to change over time, the precise frequency is much debated and remains poorly understood. Several studies report a peak in the frequency of movements during the second trimester, followed by a decrease in frequency towards full term [3–6], while other researchers find decreases in movements over gestation [7,8]. Sudden changes in fetal movements can be indicative of fetal compromise, and reduced fetal movement can signify fetal distress that necessitates urgent delivery [9,10]. Decreased fetal movements approaching term correlate with poor fetal outcomes, such as low birth weight or preterm delivery [11,12], as well as fetal death [10,13].

Fetal movements are known to play a significant role in normal musculoskeletal development (reviewed in [14]), likely because the resulting muscle forces generate stress and strain within the fetal skeleton that stimulates the developing skeletal tissues. Abnormal skeletal development has been observed clinically in cases of neuromuscular disorders that result in reduced or absent fetal movement, with patients presenting skeletal malformations such as joint fusions, craniofacial abnormalities and hypo-mineralized bones [15–17]. Fetal akinesia deformation sequence (FADS), for example, is a rare syndrome (1 : 15 000) in which fetal movement is absent [18] and leads to thin bones, multiple joint contractures, spinal abnormalities and clenched hands [19,20]. Arthrogryposis (1 : 3000), a congenital syndrome characterized by bent or abnormally angled joints in multiple body parts, and in some cases congenital scoliosis, is also associated with decreased and absent fetal movements at various gestational ages [21–26]. A relatively common joint abnormality (1.3 : 1000), known as developmental dysplasia of the hip (DDH) [27], is indicated by instability, malformation or dislocation of the joint formed at the junction of the femoral head and the acetabulum [28]. Despite known genetic risk factors for DDH, such as female gender and positive family history [29], common risk factors relate to a more restrictive uterine environment for fetal movements. Examples of these risk factors include fetal breech position [30], oligohydramnios (low amniotic fluid volume) [31] and neuromuscular disorders [29], suggesting a relationship between reduced fetal movement and abnormal hip joint development in humans [14]. Finally, metabolic bone disease of prematurity is a post-natal condition that leads to bone softening and fractures, occurring in up to 30% of extremely preterm infants (born before 28 weeks' gestation) [32]. While nutrition plays a role in this condition, the physical environment postnatally is dramatically different from the uterus, and therefore changes in biomechanical stimuli, such as stress and strain, likely contribute to its aetiology [32].

Clinical evidence for the impact of impaired fetal movement on skeletal development has been reinforced by studies of fetal mechanical stimulation in animal models, in which similar spine, bone and joint abnormalities arise in both immobilized chick embryos and mutant mouse embryos with reduced or absent muscle activity [33,34], as reviewed in [35]. A recent bioreactor study demonstrated that there is a dose-dependent relationship between movement and joint morphogenesis in the chick embryo [33]. Taken together, this evidence suggests that normal prenatal musculoskeletal development requires mechanical forces generated by active fetal movements. Further, because joint shape has been linked to the risk of osteoarthritis [36], the contribution of fetal movements to a healthy joint shape has major implications for an individual's health in later life. However, given the challenges of measuring fetal movements experimentally, little is known about the biomechanics of these movements in human babies and how they change throughout gestation.

A key step in the process of skeletal development is the transduction of mechanical stimuli into biochemical signalling that results in changes in skeletal architecture. Computational modelling provides a method by which this mechanobiological relationship can be investigated and attempts have been made to model this relationship in animals. Such studies have revealed that biomechanical stimuli correlate with cell behaviour and joint shape in zebrafish [37], with ossification of avian embryonic bones [38] and with mechanosensitive gene expression in joints of mutant mouse embryos [39]. Until recently, movement quantification and reconstruction of fetal skeletal geometry (necessary to calculate biomechanical stimuli) was not possible for human fetuses. However, advances in fetal magnetic resonance imaging (MRI), known as cine-MRI scans, now allow movements of an entire fetus to be directly observed [3,40]. This technology permits the use of computational techniques, including finite element (FE) analysis and musculoskeletal modelling, to quantify kick and associated muscle forces at a particular gestational age for the first time [41].

In this study, we build upon our previously developed fetal movement modelling techniques by applying predicted muscle forces to three-dimensional fetal skeletal geometries. We quantify the stress and strain induced in these developing skeletal structures due to clinically observed fetal movements for the first time. We find a significant upward trend in kick forces from 20 to 30 weeks' gestation, before decreasing towards term. We reveal that stress and strain increase significantly over the latter half of gestation, which is important because changes in fetal movement patterns in late pregnancy have been linked to poor fetal outcomes and musculoskeletal malformations.

## 2. Material and methods

**Figure 1.**
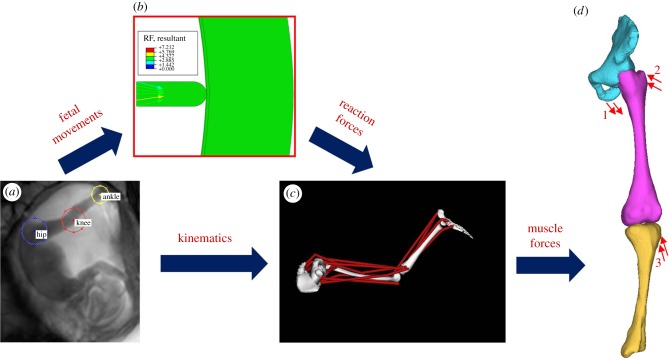
Flowchart outlining the computational pipeline developed for this study. Computational methods applied comprise (*a*) tracking of fetal joint movements, (*b*) finite element modelling of reaction force resulting from fetal movements against the uterine wall, (*c*) musculoskeletal modelling to predict muscle forces, (*d*) application of muscle forces to finite element models of fetal geometries (forces for adductor magnus (1), gluteus maximus (2) and biceps femoris (3)).

In order to quantify the stress and strain in the fetal skeleton due to kicking during pregnancy, the following pipeline of computational techniques was applied to age-varying datasets during this study: (i) tracking the fetal movements of the joints from cine-MRI scans, (ii) FE modelling of these movements to determine the resulting reaction force from the uterine wall, (iii) combining the above outputs in a musculoskeletal model which predicts the intramuscular forces required to generated the observed displacements, and (iv) application of these forces in an FE model of fetal limb geometries in order to calculate the resulting stress and strain. This computational pipeline is illustrated in figure 1 and electronic supplementary material, movie S1, and the methods are described in detail in this section.

### 2.1. Data acquisition

A database of fetal cine-MRI scans acquired from archived data at Hammersmith Hospital and St. Thomas' Hospital, London, UK, was retrospectively analysed for those which included extension–flexion fetal kicks. A total of 341 scans from different individuals were examined of which 20 were chosen in which there was a clear in-plane extension of the lower limb, in order to generate a cross-sectional study of four sub-sets of five scans at approximately 20, 25, 30 and 35 weeks' gestation (electronic supplementary material, table S1 and movie S2). All women had given prior consent for scans to be used in research as part of larger ethically approved trials (Hammersmith Hospital Research Ethics Committee/MHRA approval for IEH award 102431).

**Figure 2.**
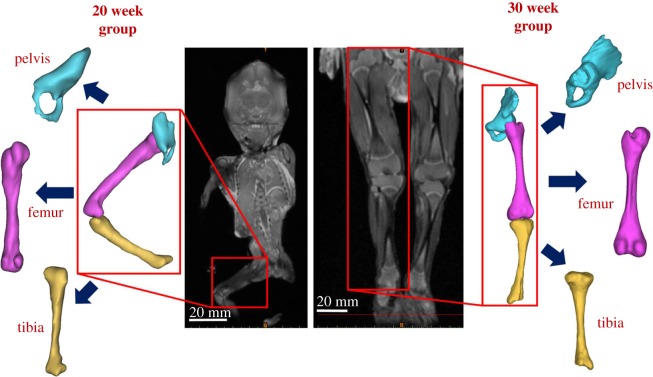
Fetal geometries obtained from post-mortem MRI. Post-mortem MRI scans at 20 and 30 weeks' gestational age allow three-dimensional reconstruction of both mineralized and cartilaginous components of the pelvis, femur and tibia.

**Figure 3.**
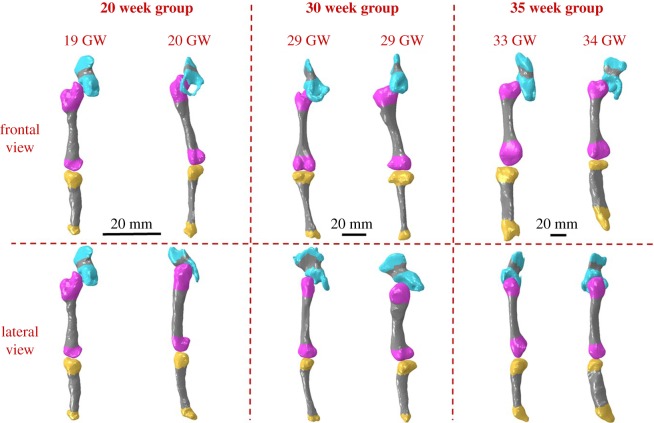
Fetal leg bone geometries grouped by gestational age. Three-dimensional geometries were reconstructed from post-mortem MRI scans, two each at approximately 20, 30 and 35 weeks. Fetal geometries increased in both size and complexity with advancing gestational age, with later gestational ages demonstrating larger, flatter iliac crests, more prominent greater trochanters and femoral condyles, and wider proximal tibia with more pronounced tibial condyles. Mineralized regions are shown in grey.

Separately, the radiology information system (RIS) at Great Ormond Street Hospital (London, UK) was searched for post-mortem MRI in fetuses between the gestational ages of 19–35 weeks, which included full anatomical imaging of the lower limbs. All parents/guardians gave written consent for pre-autopsy MRI as part of the GOSH clinical autopsy protocol, and for research use of imaging material. Cases were excluded where there was a known or suspected musculoskeletal abnormality, either on post-mortem MRI or on subsequent autopsy. Six scans were included in this study, two from approximately 20 weeks' gestation (a 19 and a 20 week), two from approximately 30 weeks' gestation (two at 29 weeks) and two from approximately 35 weeks' gestation (a 33 week and a 34 week) as shown her in figures 2 and 3. Scan settings for all data collection are detailed in the electronic supplementary material, table S2.

### 2.2. Fetal movement tracking

A custom-designed Matlab R2014b (Mathworks, UK) software script, developed and described in detail previously [41], was applied to track the movements of individual fetal joints observed in *in utero* cine-MRI data of fetal kicking (figure 1*a* and electronic supplementary material, movie S1). This tracking software was tested previously, and found to be fully repeatable and accurate in approximately 95% of cases compared to manual selection by an experienced human operator [41]. Additionally, the uterine dimensions were measured, assuming an elliptical shape with a major and a minor axis. A series of images was analysed for each fetus, capturing the kick and contact with the uterine wall, up to the point of greatest deflection of the wall.

### 2.3. Calculation of fetal kick force

In order to calculate the reaction force resulting from an *in utero* fetal kick, FE simulations of the uterine mechanical environment were developed in ABAQUS (Dassault Systemes, Vélizy-Villacoublay, France) FE software (figure 1*b*), detailed in a previous study [41]. Briefly, the uterus was modelled in two dimensions as half an ellipse, taking the two-dimensional measurements from the scans as inputs, with symmetry boundary conditions applied at the boundaries and using major and minor axis dimensions taken from each scan. Mesh convergence analyses were performed to optimize mesh density. The uterus was pre-stressed by applying the average maximum and minimum observed intrauterine pressures, as described previously and in the electronic supplementary material, table S1 [42]. Observed kick displacement from the cine-MRI was applied as ramped, static loading using a probe of the same diameter as the fetal foot. The uterine wall was modelled as a 6.0–6.8 mm thick layer of uterine muscle depending on gestational age [43]. The fetal membrane was modelled as two layers, the chorion and the amnion, with the outer surface of the chorion attached to the uterine wall, while frictionless sliding contact was assumed at the interface between the chorion and the amnion [44]. Linear elastic, isotropic behaviour was assumed for all materials, with elastic moduli, Poisson's ratios and thicknesses described in table 1 as previously [42]. These models have previously been validated experimentally [41], and a sensitivity analysis demonstrated that a 10% increase in uterine muscle stiffness resulted in a 3.5% increase in kick reaction force.

**Table 1.** Material properties and thicknesses applied in FE models for amnion and chorion [45–47], uterine wall [43,48] and fetal cartilage [49–51].

	amnion	chorion	uterine wall	unmineralized fetal cartilage	mineralized fetal cartilage
Young's modulus (MPa)	21	2.3	0.586	1.1	117
Poisson's ratio	0.4	0.4	0.4	0.49	0.49
thickness (mm)	0.044	0.188	6.0, 6.5, 6.8	—	—

### 2.4. Prediction of muscle forces generated by fetal kicks

The fetal joint movements obtained from the cine-MRI tracking software were combined with the predicted reaction forces as inputs for a scaled musculoskeletal model of the fetal lower limb in OpenSim (v. 2.4) [52], as shown in figure 1*c* and described previously [41]. The displacements of the hip, knee and ankle joints were then applied to the relevant joint markers on the musculoskeletal model, and the reaction forces from the FE models were applied at the calcaneus (heel bone) of the fetal foot. The OpenSim model was restricted to planar motion as the fetal movements selected occurred in the single plane visible in the MRI scan. Muscle forces for 19 separate muscles were outputted from OpenSim, alongside their lines of action using a previously developed plugin [53].

### 2.5. Characterization of stress and strain in the fetal skeleton

In order to investigate the biomechanics of the fetal lower limb during kicking *in utero*, sets of FE models of the fetal leg bones were generated from post-mortem MRI scans at multiple gestational ages (figure 1*d*). Geometries for the pelvis, femur and tibia were segmented using MIMICS image processing software, including mineralized and non-mineralized regions detected on the post-mortem MRI, and meshed using 4-noded tetrahedral (C3D4) elements using 3-Matic software (both Materialise, Leuven, Belgium) (figures 2 and 3). Geometries contained between 34 000 and 290 000 elements per model, with mesh refinement tools in 3-Matic used to optimize mesh quality and mesh convergence analyses performed to optimize mesh density. As no post-mortem MRI scans at 25 weeks were available, the 20 and 30 week geometries were scaled up or down according to published femoral length reference values at 20, 25 and 30 weeks [54]. These scaled geometries were then pooled to form a group of four geometries, on which the 25 week muscle forces were applied.

Fetal geometries were then imported into ABAQUS, with all materials assumed to be linear elastic and isotropic in nature. The pelvis was fixed at the pubic symphysis and the sacroiliac joint, with the femur and tibia displaced until frictionless contact was achieved at the joints. The muscle forces predicted by the musculoskeletal model at the end of the leg extension were then applied at anatomical locations (as illustrated in figure 1) and allowed to converge to equilibrium, generating stress and strain within the models. Maximum stress and strain were recorded as the 95th percentile values, to avoid potential artificial stress concentrations at the interface between the mineralized and unmineralized cartilage regions. This process was repeated for each cine-MRI movement, and on each geometry per group.

### 2.6. Statistical analysis

Kicks from cine-MRI scans of five different fetuses were analysed per gestational age group, and applied to two geometries at each of 20, 30 and 35 weeks (*n* = 30 load cases) and four scaled geometries at 25 weeks (*n* = 20 load cases). Statistical differences (in maximum force, stress and strain) between age groups were determined using an ANOVA analysis and a Tukey's post hoc test, with statistical significance defined as *p* < 0.05 (SPSS, IBM, New York, USA). All data are expressed as mean ± s.d. In order to distinguish between effects of geometry and age, statistical differences between scaled geometries at 25 weeks were determined using an independent two-tailed Student's *t*-test, with statistical significance defined as *p* < 0.05 (SPSS, IBM, New York, USA).

## 3. Results

### 3.1. Characterization of fetal skeletal morphology

Three-dimensional geometries of the lower limb generated from post-mortem MRI scans of specimens increased in size with increasing gestational age, as expected, but also demonstrated increased complexity in shape with advancing gestational age (figure 2). Notable features of morphogenesis included larger, flatter iliac crests, more prominent greater trochanters and femoral condyles and wider proximal tibia with more pronounced condyles (figure 3). Note that due to differences in size, and the settings/resolutions used according to gestational age, complex shape features were most apparent in the 30 week group.

### 3.2. Fetal muscle forces, stress and strain increase during gestation

**Figure 4.**
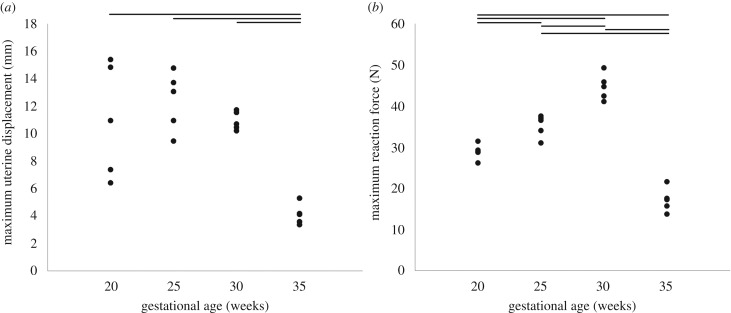
Maximum observed uterine displacements and resulting fetal kick forces. Average results for 20, 25, 30 and 35 weeks' gestational age, for (*a*) uterine wall displacement and (*b*) uterine reaction force. Horizontal lines indicate statistical significance between groups (*p* ≤ 0.05).

The average displacement of the uterine wall due to observed kicks did not change significantly between 20 and 30 weeks' gestation, remaining at approximately 11 mm (figure 4*a*, table 2 and electronic supplementary material, movie S2). However, uterine wall displacement decreased significantly at 35 weeks, to approximately 4 mm. Fetal kick force increased significantly over time, from approximately 29 to 47 N between 20 and 30 weeks (figure 4*b* and table 2), before decreasing significantly to 17 N at 35 weeks. The mean and standard deviation of these results, alongside average fetal femur and tibia lengths, uterine dimensions and kick durations, are presented in table 2.

**Table 2.** Fetal uterine parameters versus gestational age: kick duration, femur and tibia length, uterine major and minor axes, uterine wall displacement and kick reaction force. Values are presented as mean ± standard deviation.

age group	kick duration (s)	femur length (mm)	tibia length (mm)	uterine major axis (mm)	uterine minor axis (mm)	uterine wall displacement (mm)	kick reaction force (N)
20 weeks	2.65 ± 0.35	58.45 ± 9.11	56.14 ± 4.22	217.19 ± 42.74	163.03 ± 17.12	11.78 ± 4.72	28.85 ± 1.88
25 weeks	3.63 ± 0.65	56.93 ± 16.47	57.44 ± 14.01	222.18 ± 51.32	166.98 ± 47.89	12.37 ± 1.99	35.17 ± 2.41
30 weeks	2.95 ± 0.74	61.37 ± 16.03	55.92 ± 9.31	236.29 ± 21.16	178.29 ± 23.36	11.52 ± 1.47	46.64 ± 5.30
35 weeks	3.51 ± 0.49	62.68 ± 2.54	55.48 ± 3.27	219.49 ± 26.74	186.75 ± 8.51	4.09 ± 0.66	17.09 ± 2.62

**Figure 5.**
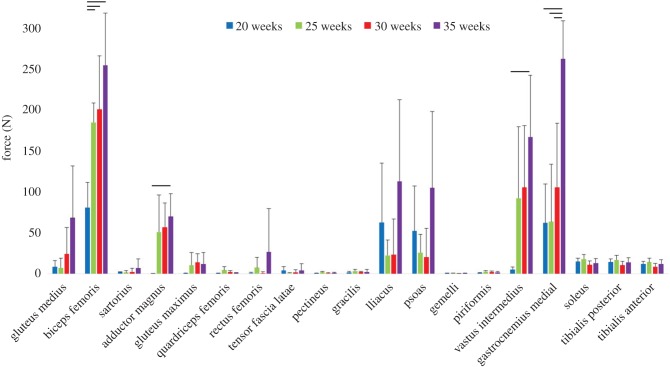
Average muscle forces at full-leg extension for 20, 25, 30 and 35 weeks' gestational age. The means and standard deviation of four groups of five kicks each are plotted; horizontal lines indicate statistical significance (*p* ≤ 0.05).

The average intramuscular force at full-kick extent is grouped by gestational age in figure 4*b*. Although there was a great deal of variation, an upward trend in muscle force during gestation was evident among many of the muscles, with statistically significant increases for the biceps femoris adductor magnus, vastus intermedius and gastrocnemius (figure 5).

**Figure 6.**
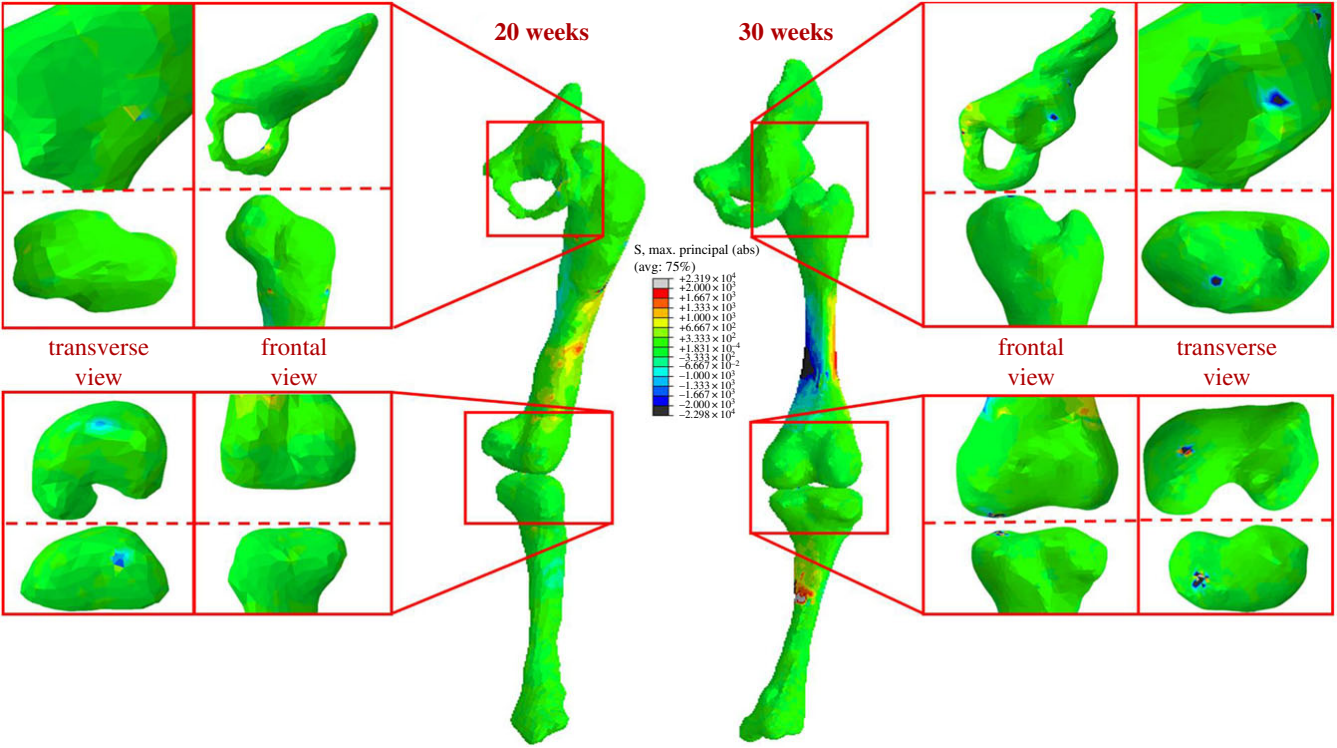
Maximum principal stress stimulation in fetal leg bones increases with gestational age. Average stress results for 20 and 30 week fetal geometries, demonstrating increased stress concentrations in mineralized regions and at joint surfaces over gestation.

**Figure 7.**
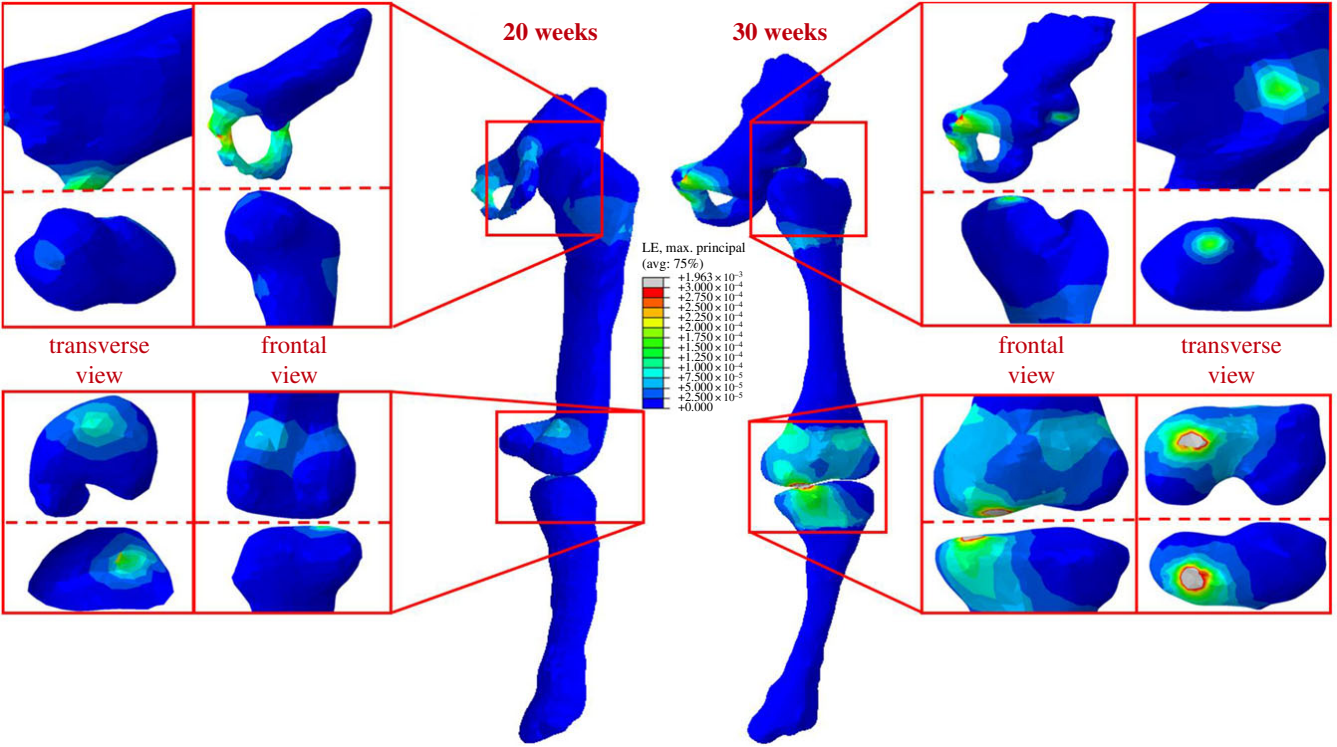
Maximum principal strain stimulation in fetal leg bones increases with gestational age. Average maximum principal strain results for 20 and 30 week fetal geometries, demonstrating increased strain concentrations in cartilage and at joint surfaces over gestation.

**Figure 8.**
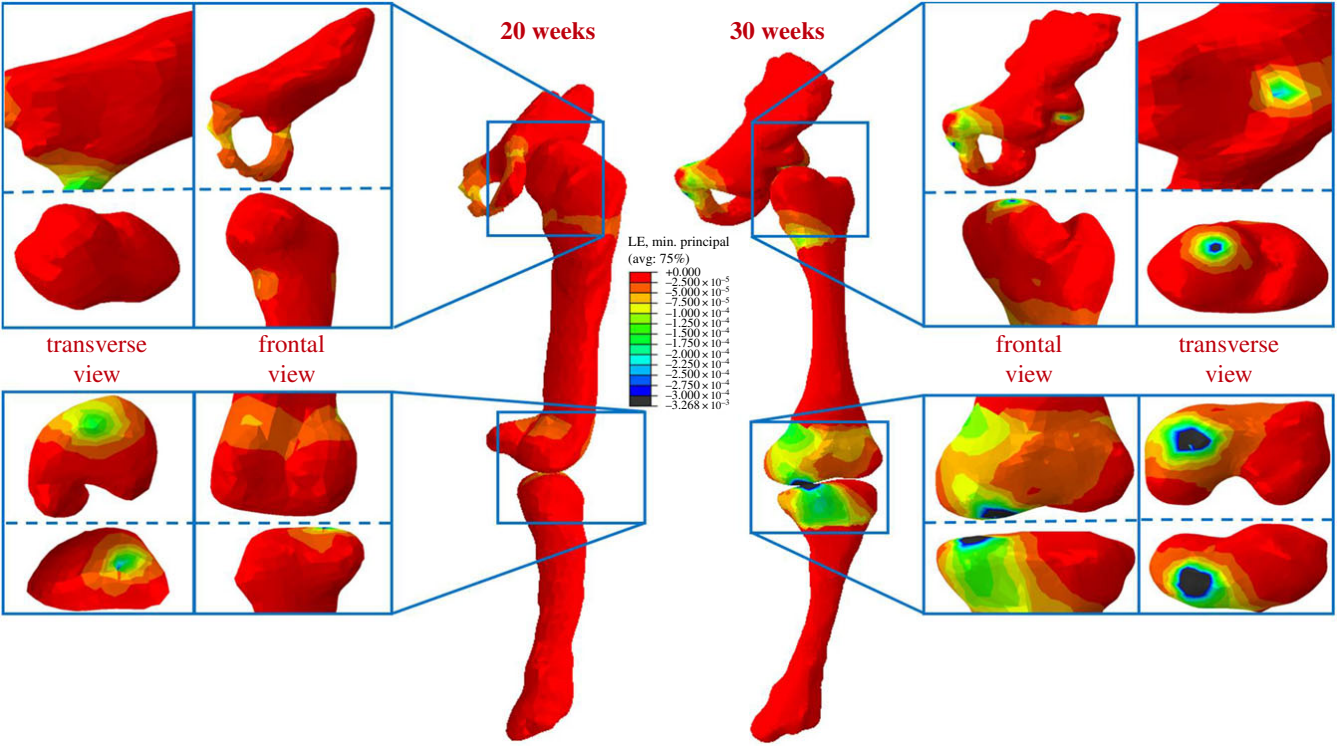
Minimum principal strain stimulation in fetal leg bones increases with gestational age. Average minimum principal strain results for 20 and 30 week fetal geometries, demonstrating increased strain concentrations in cartilage and at joint surfaces over gestation.

At all gestational ages, concentrations of maximum principal stress were observed in the shaft of the femur and tibia, and at joint surfaces where contact between each fetal bone occurred (figure 6). The greatest stresses occurred in the mineralized diaphysis regions of the bones, and at the interface of these regions with unmineralized cartilaginous regions, suggesting a link between stress and ossification during development. In contrast to stress, strain was concentrated in the unmineralized regions near the joints and at the joint surfaces at all ages (figures 7 and 8), indicating that these strains may play a role in shaping joints during development.

**Figure 9.**
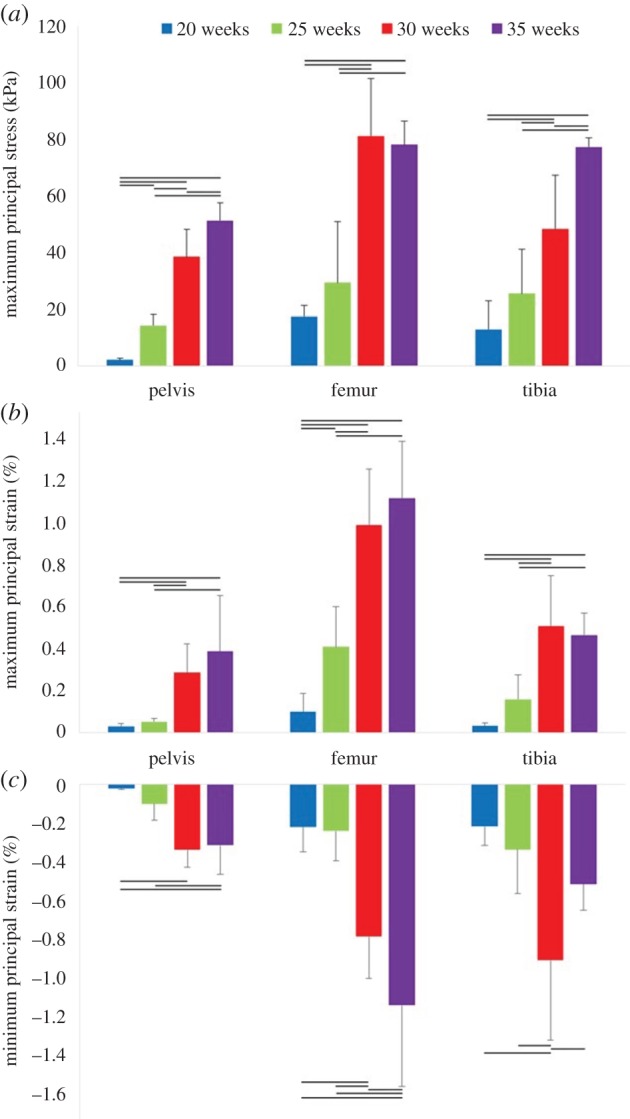
Biomechanical stress and strain in fetal leg bones over second half of gestation. Average results for 20, 25, 30 and 35 weeks' gestational age, for (*a*) maximum principal stress, (*b*) maximum principal strain, (*c*) minimum principal strain. The means and standard deviation of four groups of five kicks each are plotted. Horizontal lines indicate statistical significance (*p* ≤ 0.05).

Maximum principal stress was found to increase significantly with gestational age for the pelvis, femur and tibia, with stress noticeably increasing in all regions from 20 to 35 weeks' gestational age (figures 6 and 9*a*). Similarly, maximum and minimum principal strains increased significantly in magnitude over the second half of gestation for all regions of each rudiment, as shown in figures 7–9*b,c*.

Finally, when a statistical analysis was performed in order to investigate the effect of scaling the 20 and 30 week geometries to 25 week dimensions, with muscle forces applied from the 25 week fetal kicks, no significant difference in stress or strain results were found between the scaled 20 and 30 week groups. This suggests that geometry is not the key determinant of stress and strain over gestational age, instead implying a stronger role for fetal kick forces.

## 4. Discussion

This study represents the first quantification of changes in the biomechanics of the developing fetal skeleton due to fetal movements, revealing an upward trend in both stress and strain stimulation over the second half of gestation. We quantify significant changes in kick force and muscle forces over gestational time due to a simple extension movement. We reveal that even though older fetuses (35 weeks) deform the uterine wall much less than at younger ages, the stresses and strains in the fetal skeleton are at least as high as at earlier gestational ages. This research provides new insight into the biomechanical environment *in utero*, and the distribution of stimuli in the fetal skeleton suggests a role for stress stimulation in ossification events and for strain stimuli in joint morphogenesis.

The human uterus during pregnancy is an experimentally inaccessible closed mechanical environment, so a number of assumptions and limitations were necessary to conduct this research. While the material properties for the uterus, fetal membranes and fetal cartilage are non-linear and likely change over gestation, these values were not available in the literature [41]. In reality, the viscoelastic and hyperelastic properties would likely result in lower reaction forces, as the tissues deformed to a greater degree, though this might change with gestation as the intrauterine diameter and pressure increase. Additionally, the lack of available post-mortem MR scans at 25 weeks necessitated scaling of the 20 and 30 week groups according to fetal femur length. Nonetheless, pooling of these data does not appear to affect stimuli results as we did not find significant differences in stress or strain between these groups when scaled to femoral length of 25 weeks. While the quadratic optimization cost function applied in the musculoskeletal model is likely different for a fetal kick, it was assumed to be the same as that for an adult, due to lack of available experimental datasets, and as they appear to be a coordinated repeated motion. Finally, depending on the image resolution and scan settings used, some shape information may have been lost during segmentation, resulting in less detailed morphologies for some samples. However, we found relatively consistent shapes in each individual at similar gestational ages and, as mentioned above, observed that differences in geometry do not appear to be the key factor influencing the stresses and strains we calculated.

The stresses and strains on the fetal skeleton observed in this study likely act as biomechanical stimuli for limb development and morphogenesis, with various studies showing that biomechanical stimuli correlate with cell behaviour and joint shape in zebrafish [37], with ossification of avian embryonic bones [38] and with mechanosensitive gene expression in the limbs of mutant mouse embryos [39]. Therefore, the biomechanical stimuli characterized in this study illuminate a crucial missing link in our current understanding of human developing skeletal biomechanics and mechanobiology. Importantly, this study quantifies a baseline of normal biomechanical stimuli resulting from fetal kicking, providing new data which can be compared to stimulation in abnormal or suboptimal uterine conditions. Skeletal development is ultimately a cell-driven process, with shape and mineralization progressing as fetal tissues respond to biomechanical stimulation, such as stress and strain [38,55–58]. However, this stimulation is impossible to investigate experimentally *in utero* in humans. The patterns of stimulation observed in our models suggest a relationship between stress concentrations and progressive ossification of the fetal bones, with the highest stresses occurring in mineralized regions of the long bones and in sites of primary ossification in the pelvis. Conversely, strain levels were highest in the unmineralized regions near the joints, indicating a potential role for high strains in joint morphogenesis. These patterns of stress and strain also provide new inputs for previously developed adaptive mechanobiological models of hip joint development and DDH [59,60], supplying physiologically relevant patterns of biomechanical stimuli for the first time. Furthermore, as the field of tissue engineering has matured, researchers have attempted to mimic the natural developmental processes of chondrogenesis and endochondral ossification as a route to successful production of tissue-engineered cartilage and bone [61,62]. Our findings provide novel insights into the distribution and magnitudes of stresses and strains that may prove key to replicating developing prenatal tissue conditions *in vitro*.

Of particular interest is the clear trend of the stresses and strains increasing significantly with gestational age at multiple steps in the computational pipeline. Specifically, we observed significantly higher kick forces, an upward trend in intramuscular forces, and significantly higher stress and strain stimulation in all components of the lower limb. Interestingly, while significantly lower uterine displacement and resulting kick force were observed at 35 weeks, this did not result in decreased stress or strain stimulation. This is likely to be due to the higher muscle forces predicted, themselves the result of a more cramped fetal position when kicking in late gestation. A similar trend of increasing stress and strain with increasing developmental stage has been predicted in the embryonic chick limb [38]. The effects of absent fetal movements are clear at multiple gestational ages, as in cases of arthrogryposis and FADS [18,21,22,25]. The current study demonstrates for the first time that there is a steady increase in biomechanical stimuli over gestation, suggesting that even a period of late restricted movements, e.g. fetal breech position, could have an impact on normal skeletogenesis and increase the risk of DDH [63]. Indeed, one theory for why metabolic bone disease of prematurity (leading to weak bones, prone to fracture) occurs in severely premature neonates is that when the last trimester of development occurs outside the uterus, biomechanical stimulation of the skeleton would be substantially different to *in utero* [64]. After birth, the absence of amniotic fluid buoyancy effects means that neonates are exposed to gravitational effects and no longer have the surrounding uterine tissues to kick against, which would likely lead to very different levels and patterns of biomechanical stimulation in a preterm infant at (for example) 30 weeks, compared to a fetus of the same age and still *in utero*. Combined with the results of the current study, this suggests that higher levels of mechanical stimulation as gestation progresses are critical to normal skeletal formation, and that movements at the end of gestation, though small in magnitude, are still important for normal skeletal development.

In summary, we have quantified the biomechanics of common human fetal movements for the first time, finding increases in fetal kick forces and muscle forces, as well as stress and strain in the fetal skeleton over the second half of gestation. We have found increases in these biomechanical with advancing gestational age, providing novel insight into the biomechanical environment *in utero*. We also observed concentrations of biomechanical stimuli in the fetal skeleton, suggesting a role for stress stimulation in ossification events and for strain stimuli in joint morphogenesis. Further analysis of these observed trends in developmental biomechanics may shed new light on the link between fetal biomechanics and skeletal malformations, and provide critical novel data for future research in tissue engineering and mechanobiology.

## Supplementary Material

Movie S1

## Supplementary Material

Movie S2

## Supplementary Material

Supplementary Data for Upload.xlsx

## Data accessibility

Electronic supplementary material (tracking code and computational models) is available online at https://dx.doi.org/10.6084/m9.figshare.5630245.
