# Supplementary figures and images for: Stresses and strains on the human fetal skeleton during development
Source: J R Soc Interface. 2018 Jan 24;15(138):20170593. doi: 10.1098/rsif.2017.0593 (PMC5805961; doi:10.1098/rsif.2017.0593)

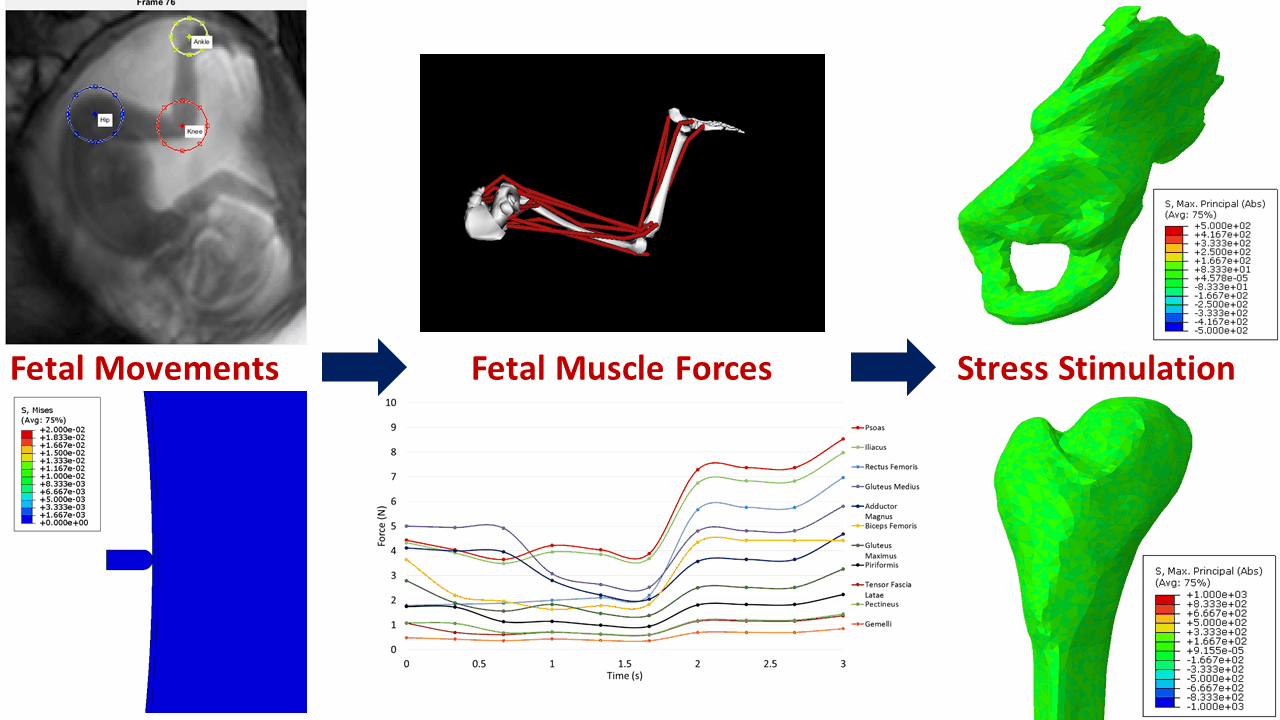

Supplement: Movie S1 [file rsif20170593supp1.gif]

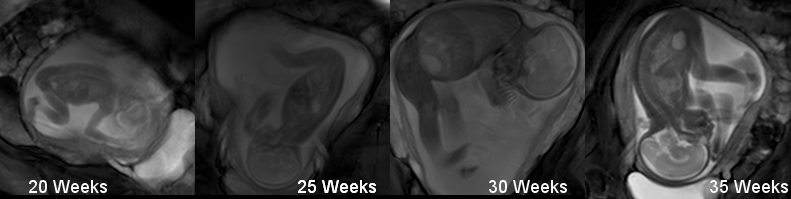

Supplement: Movie S2 [file rsif20170593supp2.gif]
